# Two sources of dopamine for the hippocampus

**DOI:** 10.1016/j.tins.2017.05.005

**Published:** 2017-07

**Authors:** Colin G. McNamara, David Dupret

**Affiliations:** 1 Medical Research Council Brain Network Dynamics Unit, Department of Pharmacology, University of Oxford, Oxford, UK

## Abstract

Dopaminergic signalling is established as playing an important role in novelty related modulation of hippocampal memory. Two recent studies have identified the noradrenergic fibres originating in the locus coeruleus as an additional source of neurotransmitter acting on dopaminergic receptors in the hippocampus.

Not all experiences are preserved as vivid memories. Details of familiar actions in familiar settings can fade instantly, yet it is often possible to remember an unusual event or a new place with remarkable clarity. For example, how often are you unsure whether you locked the door of your house, even as you attempt to recall the event just moments later walking down the street, yet you can vividly recall leaving your hotel to explore a new town during last year’s holiday? In investigations into the neural basis of this phenomenon, it was demonstrated that dopamine affects plasticity, synaptic transmission and the network activity in the hippocampal circuitry underlying this type of memory 1, 2, 3, 4. Rodent behavioural studies using pharmacological manipulation of hippocampal D1/D5 dopamine receptors provided much of the early evidence, with the source of dopamine long thought to be the ventral tegmental area (VTA) 1, 5. However, recent studies have brought the exciting insight that hippocampal dopamine can arise from two sources, and the resulting effects on hippocampal function may differ. Specifically, in addition to dopamine release from the known sparse innervation of the hippocampus by dopaminergic fibres of the VTA 1, 2, 4, 6, two publications now report that dopamine release from noradrenergic fibres of the locus coeruleus (LC) underpin memory associated signalling in the hippocampus 7, 8.

In the first, Takeuchi and colleagues used a spatial learning task in which mice daily learned a new location of a small reward hidden below the arena bedding [7]. Memory for the reward location was recalled after 1 hour but performance degraded to chance after 24 hours. However, burst optogenetic activation of the LC 30 minutes after encoding caused memory for the reward location to persist for 24 hours. This memory – which was encoded in a familiar arena – was similarly prolonged by exposing mice to a novel environment instead of LC activation, suggesting that LC activation was sufficient to mimic the memory enhancing effects of the novel experience. Curiously, this enhancement of memory by post-encoding novelty exposure was blocked by intra-hippocampal infusion of the D1/D5 receptor antagonist SCH23390 but was not altered by infusion of the β-adrenoceptor antagonist propranolol. The same result was reached when the pharmacological manipulations were applied to the optogenetic LC activation protocol. Finally, VTA activation in this paradigm failed to improve memory. These results first seemed to present a contradiction: activation of LC noradrenergic neurons produced a behavioural effect yet it required hippocampal dopaminergic receptors. The logical resolution was to conclude that LC originating noradrenergic fibres (LC-TH^+^) released dopamine in the hippocampus. The authors further showed that optogenetic activation of LC-TH^+^ fibres in hippocampal slice preparations boosts CA3–CA1 synaptic plasticity in a manner consistent with dopamine release from noradrenergic terminals but actual transmitter release remained to be measured.

More recently, Kempadoo and colleagues used high-performance liquid chromatography to measure catecholamine release into the extracellular fluid of photostimulated hippocampal slice preparations from mice expressing Channelrhodopsin-2 in LC-TH^+^ axons [8]. The authors observed a near fourfold increase in both noradrenaline and dopamine concentrations between light off and light on conditions, demonstrating dopamine release by noradrenergic fibres (with the dopamine concentration being one tenth that of noradrenaline). They also showed that optogenetic activation of LC-TH^+^ hippocampal fibres in mice performing a spatial object recognition task enhanced learning in a D1/D5 (and not β-adrenergic) receptor dependent manner. Likewise, learning across days of an escape hole located on a Barnes Maze was enhanced, yet no improvement was seen in a spatial conditioned place preference task. Unlike in the Takeuchi *et al.* study, optogenetic stimulation was delivered during learning and not delayed until after.

The effect of LC-TH^+^ inputs on hippocampal firing dynamics associated with learning and memory remain to be ascertained. Recently, it was found that optogenetic activation of dopaminergic VTA fibres (VTA-TH^+^) in the mouse dorsal hippocampus during single day learning on the crossword maze enhanced memory retention 1 hour later [4]. In the intervening post-learning sleep period off-line reactivation of hippocampal firing patterns associated with that learning was enhanced and later, during the memory test, the place cell population map activity expressed during learning was better reinstated. Assessing the effect of LC-TH^+^ activation on hippocampal firing dynamics and comparing it to VTA-TH^+^ activation could prove a fruitful approach in contrasting the functional contribution of these two neuronal pathways.

Both the Takeuchi *et al.* and Kempadoo *et al.* work highlighted the long standing finding that LC-TH^+^ fibres more densely innervate the rodent dorsal hippocampus, with Kempadoo *et al.* quantifying LC-TH^+^ fibre density to be 4.5 times that of VTA-TH^+^ fibres. Potentially, VTA-TH^+^ fibres may selectively target particular hippocampal synapses or neuronal cell types – such as a particular population of interneurons – allowing for a targeted yet far-reaching effect, whereas LC-TH^+^ fibres may release dopamine non-selectively, resulting in a more widespread modulatory action. Alternatively, the specialised dopaminergic VTA-TH^+^ fibres could be just as efficient at releasing dopamine, given that measured dopamine concentration from LC-TH^+^ fibres was a fraction of noradrenaline release from the same terminals [8]. Finally, an intriguing possibility is that a key aspect of dopamine action could lie in its co-release with other chemical partners.

In any case, converging evidence from optogenetic activation of these distinct projections during behaviour suggests differences in the temporal specificity of their effect: activation of LC-TH^+^ neurons 30 minutes after a learning event enhanced memory for the earlier event – in keeping with LC’s acknowledged role in regulating widespread alertness – whereas VTA-TH^+^ stimulation in the same protocol did not produce a significant memory enhancement [7]. Conversely, VTA-TH^+^ stimulation applied during learning increased memory retention and enhanced off-line reactivation of the concurrently expressed hippocampal firing patterns, but did not extend its effect back to recently expressed patterns, in keeping with the VTA’s role in more tightly instructing learning based on value [4]. Interestingly, despite the aforementioned differences, it should be noted that both VTA and LC catecholaminergic neurons showed sustained increased firing during exposure to spatial novelty without any direct association with a discrete reward 4, 7. The LC-TH^+^ system may be optimised to produce a more temporally widespread modulation of hippocampal plasticity while the VTA-TH^+^ system may be more important for modulating plasticity to enhance cross-area neuronal representations and coordination of their stabilisation 4, 9, 10, 11. Both systems may act through similar signalling mechanisms in the hippocampus, since both act in a D1/D5 receptor dependent manner 4, 7, 8. While disentangling the effects of these two sources of dopaminergic signalling on hippocampal network dynamics remain a task for the future, the studies by Takeuchi *et al.* and Kempadoo *et al.* have established the LC as an important source of dopaminergic signalling in novelty related hippocampal learning.
